# “To have, to hold, from this day forward”: understanding current practice regarding the retention of trial participants

**DOI:** 10.1186/1745-6215-16-S2-P104

**Published:** 2015-11-16

**Authors:** Anne Daykin, Athene Lane, Carrol Gamble, Anna Kearney, Jane Blazeby, Mike Clarke, Paula Williamson, Ali Heawood

**Affiliations:** 1 University of Bristol, Bristol, UK; 2 University of Liverpool, Liverpool, UK; 3 Queen's University, Belfast, UK

## Background

Inadequate retention can introduce bias and reduce the power of a trial. The aim of this qualitative study was to elicit detailed accounts from trialists regarding strategies used to enhance retention in clinical trials.

## Methods

A purposive sample of five trials was selected from the Health Technology Assessment Portfolio of currently funded trials. Semi-structured interviews explored methods utilised by trial teams when collecting data and retaining participants. The data was analysed thematically using techniques of constant comparison.

## Findings

Clear and cohesive definitions of retention were given. However, there was less agreement about the concept of ‘withdrawal’ from a trial. More experienced trialists emphasised different levels of withdrawal and were happy to negotiate with participants in order to at least collect primary outcome data. Novice trialists presumed the participants wanted to withdraw from all aspects of the trial and made no further contact with them.

Research Nurses used their interpersonal skills to motivate participants to remain in their trials. This required time not routinely acknowledged within the funding of trials.

Participants described proactive and reactive approaches to retention. Proactive approaches involved anticipated and considered strategies, both formal and informal, to maintain retention. Conversely, reactive approaches were typified by unanticipated and spontaneous strategies, some formalised others informal, developed in response to retention problems during the trial.

## Conclusion

The experience of trialists influences their actions during the retention of patients. Recommendations from this study highlight the need for sharing proactive approaches to retention that will benefit the trials community.

